# Development of PET Imaging to Visualize Activated Macrophages Accumulated in the Transplanted iPSc-Derived Cardiac Myocytes of Allogeneic Origin for Detecting the Immune Rejection of Allogeneic Cell Transplants in Mice

**DOI:** 10.1371/journal.pone.0165748

**Published:** 2016-12-08

**Authors:** Noriyuki Kashiyama, Shigeru Miyagawa, Satsuki Fukushima, Takuji Kawamura, Ai Kawamura, Shohei Yoshida, Akima Harada, Tadashi Watabe, Yasukazu Kanai, Koichi Toda, Jun Hatazawa, Yoshiki Sawa

**Affiliations:** 1 Dept. Cardiovascular Surgery, Osaka University Graduate School of Medicine, Osaka, Japan; 2 Dept. Nuclear Medicine and Tracer Kinetics, Osaka University Graduate School of Medicine, Osaka, Japan; 3 PET Molecular Imaging Center, Osaka University Graduate School of Medicine, Osaka, Japan; 4 Dept. Molecular Imaging in Medicine, Osaka University Graduate School of Medicine, Osaka, Japan; 5 Immunology Frontier Research Center, Osaka University, Osaka, Japan; Wayne State University, UNITED STATES

## Abstract

Allogeneic transplantation (Tx) of induced pluripotent stem cells (iPSCs) is a promising tissue regeneration therapy. However, this inevitably induces macrophage-mediated immune response against the graft, limiting its therapeutic efficacy. Monitoring the magnitude of the immune response using imaging tools would be useful for prolonging graft survival and increasing the therapy longevity. Minimally invasive quantitative detection of activated macrophages by medical imaging technologies such as positron emission tomography (PET) imaging targets translocator protein (TSPO), which is highly expressed on mitochondrial membrane, especially in activated macrophage. *N*,*N*-diethyl-2-[4-(2-fluoroethoxy) phenyl]-5,7-dimethylpyrazolo[1,5-*a*]pyrimidine-3-acetamide (DPA-714) is known as a TSPO ligand used in clinical settings. We herein hypothesized that immune rejection of the transplanted iPSC-derived cardiomyocytes (iPSC-CMs) of allogeneic origin may be quantitated using ^18^F-DPA-714-PET imaging study. iPSC-CM cell-sheets of C57BL/6 mice origin were transplanted on the surface of the left ventricle (LV) of C57BL/6 mice as a syngeneic cell-transplant model (syngeneic Tx group), or Balb/c mice as an allogeneic model (allogeneic Tx group). ^18^F-DPA-714-PET was used to determine the uptake ratio, calculated as the maximum standardized uptake value in the anterior and septal wall of the LV. The uptake ratio was significantly higher in the allogeneic Tx group than in the syngeneic group or the sham group at days 7 and day 10 after the cell transplantation. In addition, the immunochemistry showed significant presence of CD68 and CD3-positive cells at day 7 and 10 in the transplanted graft of the allogeneic Tx group. The expression of TSPO, *CD68*, *IL-1* beta, and *MCP-1* was significantly higher in the allogeneic Tx group than in the syngeneic Tx and the sham groups at day 7. The ^18^F-DPA-714-PET imaging study enabled quantitative visualization of the macrophages-mediated immune rejection of the allogeneic iPSC-cardiac. This imaging tool may enable the understanding and monitoring host-immune response of the host, allogeneic cell transplantation therapy.

## Introduction

Cardiomyocytes (CMs) derived from induced pluripotent stem cells (iPSC) have been reported to be a promising cell source for cardiac regenerative therapy [1, 2]. iPSC derived CMs of allogeneic origin meet the clinical need to treat heart failure, such as ready to use graft. However, host immune response against the graft is of concern because it impairs the survival of the grafted cells and thus limits the therapeutic efficacy and longevity [3, 4]. A number of the strategies/treatments are under development to reduce the immunogenicity of the iPSCs and their derivatives. These include using iPSCs from donors with homologous major histocompatibility complex, modification of immunosuppressive drug treatment or supplementation of regulatory immune cells [5, 6]. Monitoring the immune rejection is poorly established in the allogeneic cell transplantation therapy for the heart, since biopsy of the transplanted graft carries substantial risks [7].

Recently developed medical imaging technologies have enabled minimally invasive quantitative detection of immune reactions using positron emission tomography (PET) imaging [8, 9]. In particular, translocator protein (TSPO) present in the outer membrane of the mitochondria and is highly expressed in the activated macrophages has been used as amarker of inflammation by using radioisotope-conjugated selective TSPO ligand, *N*,*N*-diethyl-2-[4-(2-fluoroethoxy)phenyl]-5,7-dimethylpyrazolo[1,5-*a*] pyrimidine-3-acetamide (DPA-714) as a PET tracer [10]. Since activated macrophages are involved in host immune response are known to accumulate in the transplanted graft, we herein hypothesized that immune rejection of the allogeneic iPSC-derived cardiac cell transplant may be quantitatively visualized by ^18^F-DPA-714-PET imaging study.

## Materials and Methods

Animal care complied with the “Guide for the Care and Use of Laboratory Animals” (National Institutes of Health publication). Experimental protocols were approved by the Ethics Review Committee for Animal Experimentation of Osaka University Graduate School of Medicine (reference number; 25-025-031).

### Cardiomyogenic differentiation of murine iPSCs and cardiac cell-sheet generation

*Luciferase* was, as described previously [11], transduced into murine iPS (miPS) cell line, 959A2-1, which was generated from C57BL/6 (B6) mouse embryonic fibroblasts by introducing Oct3/4, Sox2, Klf4, and c-Myc without viral vectors [12]. The iPSCs were cultured without serum or feeder cells by using ESGRO Complete PLUS Clonal Grade Medium (Millipore, Waltham, MA). Cardiomyogenic differentiation of the luciferase-expressing iPSCs was performed as described previously, followed by purification by using glucose-free medium supplemented with lactic acid [13].

**Fig 1:**
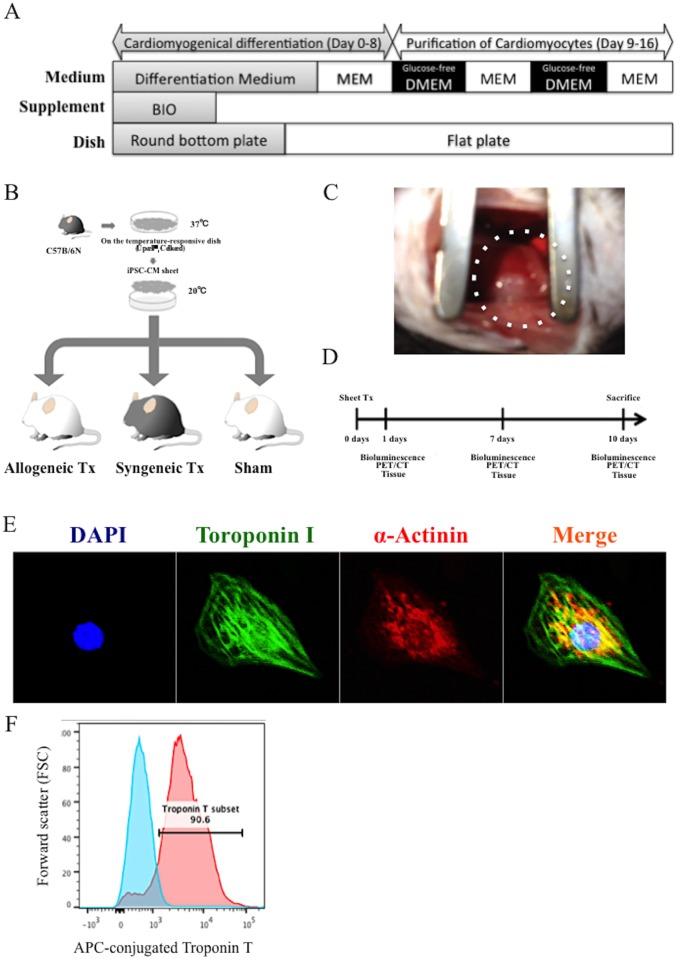
Protocol for the cardiomyogenic differentiation of murine iPSCs and cardiac cell-sheet generation, and transplantation of the cell-sheet into murine model. A, the protocol for cardiomyogenic differentiation and purification of murine iPSCs; B, C57BL/6 mice-derived iPSC-cardiac sheet was transplanted over the LV surface of the C57BL/6 mice or the Balb/c mice, as the syngeneic or the allogeneic cell transplantation models, respectively. Sham operation, thoracotomy and pericardiotomy, was performed in the Balb/c mice; C, murine iPSC-cardiac sheet transplantation to the surface of LV via left thoracotomy, dotted line indicates iPSC-cardiac cell sheet; D, the protocol for evaluation by bioluminescence imaging, ^18^F-DPA-714 PET/ CT imaging, autoradiography, or immunohistochemistry of the cardiac tissue for immunochemical histology; E, iPSC-CMs stained with anti-alpha-actinin antibody (Alexa Fluor 647), anti-troponin I (Alexa Fluor 488) and DAPI, were analyzed by confocal laser scanning microscopy; F, iPSC-CMs stained with Alexa Fluor 647-conjugated anti-troponin T antibody or the isotype control were analyzed by flow cytometry.

Briefly, the iPSCs were re-suspended in 100-mL aliquots of differentiation medium (DM), in which 100 mmol/L non-essential amino acids (Invitrogen, Carlsbad, CA), 2 mmol/L ^L^-glutamine (Invitrogen), and 0.1 mmol/L 2-mercaptoethanol (Invitrogen), and 0.2 mmol/L 6-bromoindirubin- 39-oxime (BIO; a glycogen synthase kinase-3b inhibitor, Calbiochem, La Jolla, CA) were added into Dulbecco’s Modified Eagle’s Medium (DMEM, Nacalai Tesque, Kyoto, Japan) containing 15% fetal bovine serum (FBS; Biofill, Victoria, Australia). The cells were cultured for 3 days in 96-well Corning Costar Ultra-Low attachment multiwell plates (Sigma-Aldrich, St. Louis, MO). On day 3, an additional 100 μL DM without BIO was added to each well. On day 5, individual embryoid bodies (EBs) were transferred to 100 mm gelatin-coated dishes. On days 6, 7, 10, 11, 14, and 15, the culture medium was replaced with serum-free Modified Eagle’s Medium (Invitrogen) with insulin transferrin-selenium-X (Invitrogen). On days 8, 9, 12, and 13, the medium was replaced with Glucose-free DMEM (Invitrogen) supplemented with 4 mmol/L lactic acid (Wako Pure Chemical, Osaka, Japan) for purification of cardiomyocytes (Fig 1A). On day 16, the contracting cell clusters were collected and dissociated using StemPro Accutase Cell Dissociation Reagent (Invitrogen) and seeded onto 24-well UpCell dishes (CellSeed, Tokyo, Japan). Two days later, the dish was incubated at room temperature to induce spontaneous detachment of the miPSC-cardiac sheet from the dish.

### miPSC-cardiac sheet transplantation into murine model

Mice were anesthetized by isoflurane inhalation and endotracheal intubation. miPSC-cardiac sheets were transplanted via left thoracotomy over the surface of the LV of adult male C57BL/6 mice (8–10 weeks old, 20–25 g, CLEA, Tokyo, Japan) as a syngeneic cell-transplant model (syngeneic Tx group, n = 15), or adult male Balb/c mice (8–10 weeks old, 20–25 g, CLEA) as an allogeneic model (allogeneic Tx group, n = 16). To determine the degree of macrophage-mediated inflammation resulting from surgery alone, Balb/c mice (n = 15) subjected to thoracotomy and pericardiotomy, and Balb/c mice without thoracotomy (n = 3) served as surgical and non-surgical controls (Fig 1B and 1C). Mice were sacrificed on days 1, 7, and 10 (syngeneic models: n = 4 at day1, n = 5 at day 7, and n = 6 at day 10; allogeneic models: n = 3 at day1, n = 10 at day 7, and n = 3 at day 10; sham models: n = 6 at day1, n = 6 at day 7, and n = 3 at day 10). miPSC-cardiac sheets were also transplanted into the dorsal subcutaneous space in Balb/c mice (n = 3). All surgeries and sacrifices were performed under deep anesthesia with isoflurane enough to minimize the animal suffering.

### Flow cytometry

miPSCs that were cardiomyogenically differentiated were dissociated with 0.25% trypsin-EDTA, fixed with CytoFix fixation buffer (BD, Franklin Lakes, NJ) for 20 minutes, permeabilized with Perm/Wash buffer (BD) at room temperature for 10 min, and then incubated with mouse antitroponin T antibody (Thermo Scientific, Waltham, MA) for 30 min. The labeled cells were washed with Perm/Wash buffer prior to incubation with the Alexa Fluor 647 rabbit anti-mouse IgG secondary antibody (Invitrogen) at room temperature for 30 min, and then assayed by using a FACS Canto II (BD) followed by Flowjo software (Tree Star, Ashland, OR) analysis.

### Radiochemistry (Radio synthesis of ^18^F-DPA-714)

Synthesis of ^18^F-DPA-714 was performed as described previously [14] (S1 Fig). Briefly, ^18^F-Fluoride in ^18^O-H_2_O was transferred to a UG-M1 synthesizer and passed through an Accell Light QMA cartridge. Trapped ^18^F-fluoride was eluted from the Sep-Pak cartridge and transferred to the reaction vessel with an eluent solution containing 33 mM K_2_CO_3_ (in 200 μL of water), acetonitrile (700 μL), and 23.3 mg of Kryptofix-222. After the reaction mixture was evaporated to dryness, 10 mg of tosylate precursor in acetonitrile (900 μL) was added to the reaction vessel (including ^18^F-KF with Kryotofix-222). The reaction vessel was heated at 85°C for 5 min for ^18^F fluorination. After cooling, the crude mixture was injected into Xterra Prep MS C18 10 μm (7.8 × 300 mm) semi-preparative reversed-phase HPLC column with a mobile phase of 0.1 M aqueous ammonium acetate (NH_4_OAc) and CH_3_CN (40:60, v/v) at a flow rate of 4.0 mL/min. The radioactive fraction corresponding to ^18^F-DPA-714 was collected and evaporated under vacuum with a rotary evaporator. The residue was reconstituted in sterile water (5 mL) and filtered through a 0.22-μm Millipore Millex GV sterile filter into a sterile pyrogen-free evacuated vial. The radioactivity obtained was 3–5 GBq, with a specific activity of 800–1500 GBq/μmol at the end of the synthesis (60–70 min from the completion of the ^18^F production). The radiochemical purity was greater than 97%.

### [^18^F]-DPA-714-PET/ CT imaging

The mice were anesthetized and the ^18^F-conjugated selective TSPO ligand, ^18^F-DPA-714 (10 MBq/0.1–0.2 ml), was injected into the tail vein. Thirty min after the injection, the mice were imaged with PET-CT (Inveon small animal scanner, Siemens, Berlin, Germany) [15]. The exact position of the LV was determined by CT imaging prior to PET imaging. After the CT scanning was complete, the mouse bed was translated axially and centered within the PET detector ring. The PET scan was acquired for 10 minutes. This duration for static PET imaging was selected based on the previous study when the whole body distribution became stabilized [16]. The PET images were reconstructed using OSEM3D-MAP (Matrix size; 256*256) by a software module of the scanner. Since the mouse remains in the same position on the bed for both PET and CT acquisitions and the relative positions of the fields of view of the PET and CT are known, the software module can use the positional information to fuse and coregister the PET and CT data. Fused images were converted to DICOM format and analyzed with OsiriX shareware (Geneva, Switzerland; www.osirixviewer.com) by manually selecting regions of interests (ROIs) as the anterior wall and the septal wall of the LV for calculation of the maximum standard uptake values (SUVmax), which were calculated as follows: SUV = radioactivity in ROI (Bq/cm^3^) / injected dose (Bq) / body weight (g). To minimize the effects of injection errors, the uptake ratio was used to compare SUVmax amongst the different models and defined as follows; Uptake ratio = (SUVmax in the anterior LV wall)/ (SUVmax in the septal LV wall). ^18^F-DPA714-PET/ CT imaging was performed on days 1, 7, and 10 (Fig 1D).

### Autoradiography

To validate the data obtained by PET imaging, animals were sacrificed immediately after PET scanning, and hearts were excised. High-resolution autoradiography was performed and analyzed by using Image J (National Institutes of Health, Bethesda, Maryland). In short, hearts were snap-frozen and sliced into 16-μm thick cryosections, and the radioactivity of the mid-cardiac slice including whole myocardium and transplanted graft was meatured overnight. Autoradiography was performed at days 1, 7, and 10.

### Bioluminescence imaging

Transplanted cell survival was monitored at days 1, 7, and 10 days with BLI using IVIS Lumina II (PerkinElmer, Waltham, MA). In brief, 6 min prior to measurement of luminescence, Rediject D-Luciferin Ultra Bioluminescence Substrate (PerkinElmer) was administered intraperitoneally at a dose of 150 mg/kg body weight. Animals were placed in a light-tight chamber, and photons emitted from luciferase-expressing cells were collected with an integration time of 5 min for each image. All images were analyzed with Living Image Software (PerkinElmer).

### Histology and immunohistolabeling

Dissociated miPSC-CMs were cultured on 4-well Lab-TekII chamber slides (Thermo Scientific) and fixed with 4% paraformaldehyde. The cells were reacted with the following primary antibodies: mouse anti-alpha-actinin antibody (Sigma-Aldrich) and rabbit anti-troponin I antibody (Abcam, Cambridge, United Kingdom), and then visualized by the following secondary antibodies: Alexa Fluor 647 donkey anti-mouse IgG and Alexa Fluor 488 goat anti-rabbit IgG (Invitrogen). The cell nuclei of the cells were stained with 4’, 6-diamidino-2-phenylindole dihydrochloride (DAPI) and then assessed by a confocal laser scanning microscopy FV1200 (Olympus, Tokyo, Japan). The heart specimens with transplanted miPSC-CMs were recovered at days 1, 7, 10 after transplantation, fixed with 10% buffered formalin and embedded in paraffin. Serial paraffin-embedded sections of 5-μm thickness were deparaffinized in xylene, dehydrated in graded ethanol mixtures, and processed for antigen retrieval by autoclaving in 0.01 M citrate buffer. The sections were immersed in methanol containing 3% hydrogen peroxide and then incubated with rabbit anti-CD68 antibody (ab125047, Abcam) and rabbit anti-CD3 antibody (clone SP7, ab116669, Abcam) for immunohistological examination. Subsequently, the sections were incubated with a biotinylated anti-rabbit IgG antibody (DAKO, Glostrup, Denmark), further incubated with a peroxidase-conjugated streptavidin (GE Healthcare, Little Chalfont, United Kingdom), and then visualized by biphenyl-3,30,4,40-tetramine (DAB) solution (Wako Pure Chemical). Slides were scanned using a Biorevo BZ-9000 (Keyence, Osaka, Japan), and the counts of positive cells were counted in 3 to 4 high-power fields (HPFs) per slide.

### Reverse-transcription and quantitative polymerase chain reaction

Total RNA was extracted from 1 mm slices from the central part of the excised hearts that included transplanted iPSC using the RNeasy RNA Fibrous Tissue Mini Kit (Qiagen, Hilden, Germany) and reverse transcribed into cDNA using TaqMan reverse transcription reagents (Applied Biosystems). Expression profiles of the following genes were examined by quantitative real-time polymerase chain reaction with the ABI PRISM 7700 (Applied Biosystems) system: interleukin*(IL)-1β* (Mm00434228_m1), *IL-2* (Mm00434256_m1), *IL-4* (Mm00445259_m1), *IL-10* (Mm00439614_m1), *monocyte chemotactic protein (MCP)-1* (Mm00441242_m1), *interferon (IFN)-γ* (Mm01168134_m1), *nitric oxide synthase (NOS)2* (Mm00440502_m1), *arginase (Arg)1* (Mm00475988_m1), *mannose receptor (Marc)1* (Mm01329362_m1), *CD68* (Mm03047343_m1), and *glyceraldehyde-3-phosphate dehydrogenase (GAPDH)* (Mm99999915_g1). The average copy number of gene transcripts was normalized to that of *GAPDH* for each sample in duplicate experiments. Relative quantity of the expression was calculated and compared among the experimental groups.

### Statistical analysis

Continuous values are expressed as the mean ± standard deviation and compared using Wilcoxon signed-rank test. Correlations between continuous variables were tested with Pearson correlation coefficient (r). A probability value <0.05 was considered statistically significant. JMP software 10.2.2 (SAS Institute, Cary, NC) was used for all statistical analyses.

## Results

### Generation of highly purified cardiomyocytes from murine iPSCs

Cardiomyogenic differentiation was successfully induced in murine iPSCs by using the well-established culture protocol [13]. Cardiomyocytes in the cell-clusters that were cardiomyogenically differentiated from murine iPSCs displayed a spindle shaped appearance and well-aligned sarcomere structures in the cytoplasm as demonstrated by immunohistological labeling of alpha-sarcomeric actinin and troponin I (Fig 1E). Cardiomyogenic differentiation efficiency of the murine iPSC was evaluated by flow cytometry analysis. More than 90% of the differentiated cells were positive for troponin T (Fig 1F).

### ^18^F-DPA-714 detectable by PET-CT

**Fig 2:**
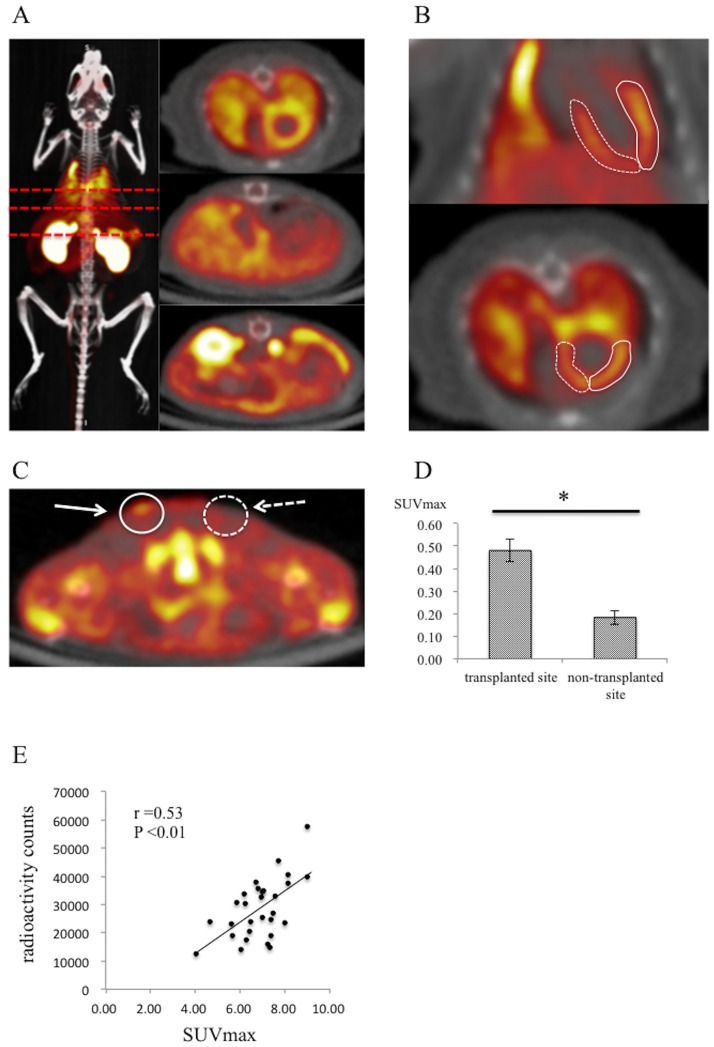
^18^F-DPA-714 detectable by PET-CT imaging. A, left side: systemic distribution of ^18^F-DPA-714 injected into tail vein was assessed in the normal Balb/c mice by PET-CT imaging, right side: ^18^F-DPA-714 PET imaging of transverse plane at the level of each red dotted lines; B, detection by PET/CT imaging of physiologic accumulation of ^18^F-DPA-714 in the anterior (solid line) and septal (dotted line) wall in LV in the normal Balb/c mice; C, white solid arrow indicates the site of transplantation of allogeneic iPSC-CMs in the dorsal side of subcutaneous space in Balb/c mice; dotted arrow indicates non-transplanted site; D, comparison of the mean SUVmax obtained by ^18^F-DPA-714 PET imaging in the transplanted site and contralateral side of subcutaneous tissue; E, correlation between SUVmax in the heart obtained by ^18^F-DPA-714 PET imaging and radioactivity obtained by autoradiography; DPA714, * indicates P <0.01.

Systemic distribution of ^18^F-DPA-714 injected into the tail vein was assessed in the normal Balb/c mice by PET-CT imaging (n = 3). Nonspecific and physiological distribution of the ^18^F-DPA-714 was clearly visualized in the heart, lung, liver, kidney, gallbladder, small intestine and bladder as reported previously [16, 17], with a mean uptake ratio of 1.08±0.04 in the heart (Fig 2A and 2B). Scaffold-free iPSC-cardiac sheet that was generated from the cell-clusters, of which 90% were troponin T-positive cardiomyocytes, was transplanted over the dorsal side of the subcutaneous space to determine the SUVmax of the iPSC-cardiac sheet. The SUVmax of the iPSC-cardiac sheet graft was significantly higher than that of the contralateral subcutaneous tissue (0.48±0.05 vs 0.18±0.03, *P* <0.01, Fig 2C and 2D). Furthermore, the SUVmax in the LV of the normal mice, intravenously injected with ^18^F-DPA-714 assessed by ^18^F-DPA-714 PET imaging and radioactivity, as assessed by autoradiography, showed significant positive correlation (n = 29, r = 0.53, *P* <0.01, Fig 2E).

### Accumulation of ^18^F-DPA-714 after the allogeneic cardiac sheet transplantation

**Fig 3:**
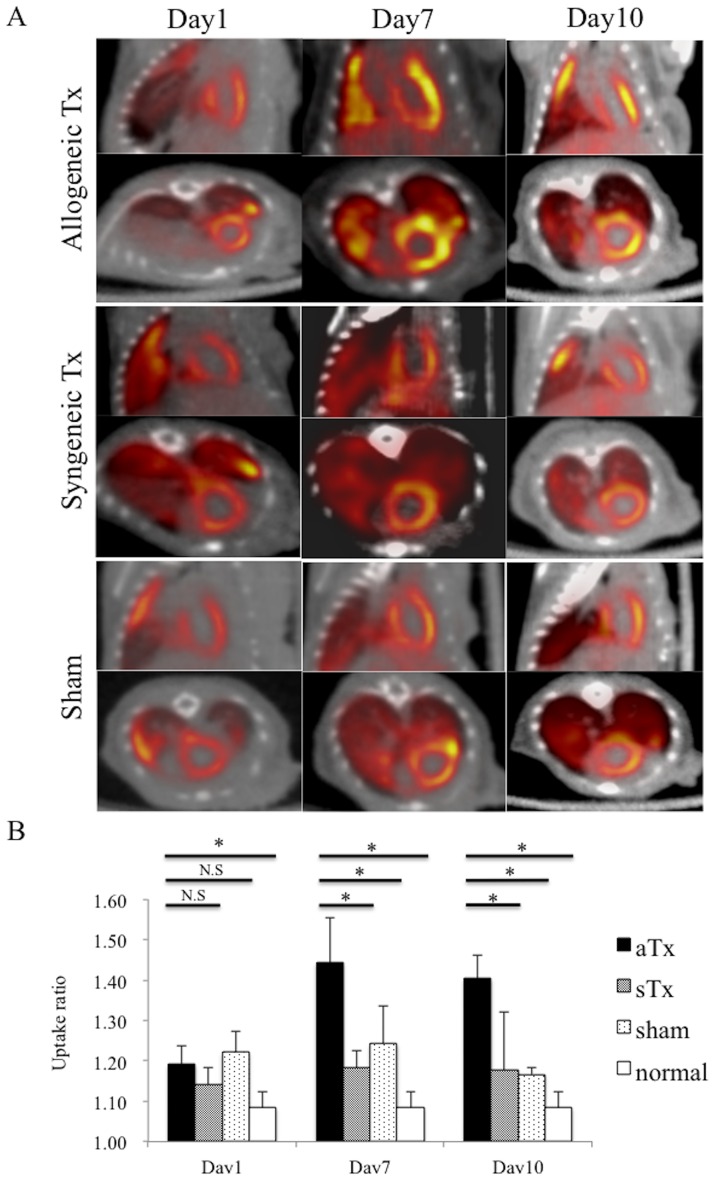
Accumulation of ^18^F-DPA-714 after the allogeneic iPSC-Cardiac sheet transplantation. A, ^18^F-DPA-714 PET/ CT imaging in the allogeneic Tx group, the syngeneic Tx group, and sham group at days 1, 7, and 10 after iPSC-cardiac sheet transplantation on the surface of LV; B, comparing uptake ratio among the allogeneic Tx group, the syngeneic Tx group, sham group and non-surgical control group at days 1, 7, and 10 after iPSC-cardiac sheet transplantation; * indicates *P* <0.05 N.S, not significant.

C57BL/6 mice-derived miPSC-cardiac sheet was transplanted over the LV surface of the C57BL/6 mice or the Balb/c mice, as the syngeneic or the allogeneic cell-transplantation models, respectively. In addition, sham operation, thoracotomy with pericardiotomy only, was performed in the Balb/c mice. The signal of intravenously injected ^18^F-DPA-714 was more prominent at day 7 and day 10 compared to day 1, in the LV of the allogeneic Tx group, in particular, in the anterior/lateral side of the LV where the cardiac sheet was transplanted. In contrast, the signal was homogeneously distributed in the LV of the syngeneic Tx group and the sham group at days 1, days 7, and 10 (Fig 3A).

The uptake ratio, calculated by computer-based quantitative analysis, was not significantly different among the groups at day 1 (syngeneic Tx group; 1.14±0.04, allogeneic Tx group; 1.19±0.05, sham group; 1.22±0.05, *P* = 0.34), whereas at day 7 and day 10, the uptake ratio was significantly higher in the allogeneic Tx group (1.44±0.11 and 1.40±0.06, respectively) than the syngeneic group (1.18±0.04, p = 0.01 and 1.18±0.14, p = 0.028 respectively), the sham group (1.19±0.14, p = 0.018 and 1.17±0.02, p = 0.046, respectively) or non-surgical control (1.08±0.04) (Fig 3B).

### Infiltration of activated macrophages and T lymphocytes into the transplanted graft

**Fig 4:**
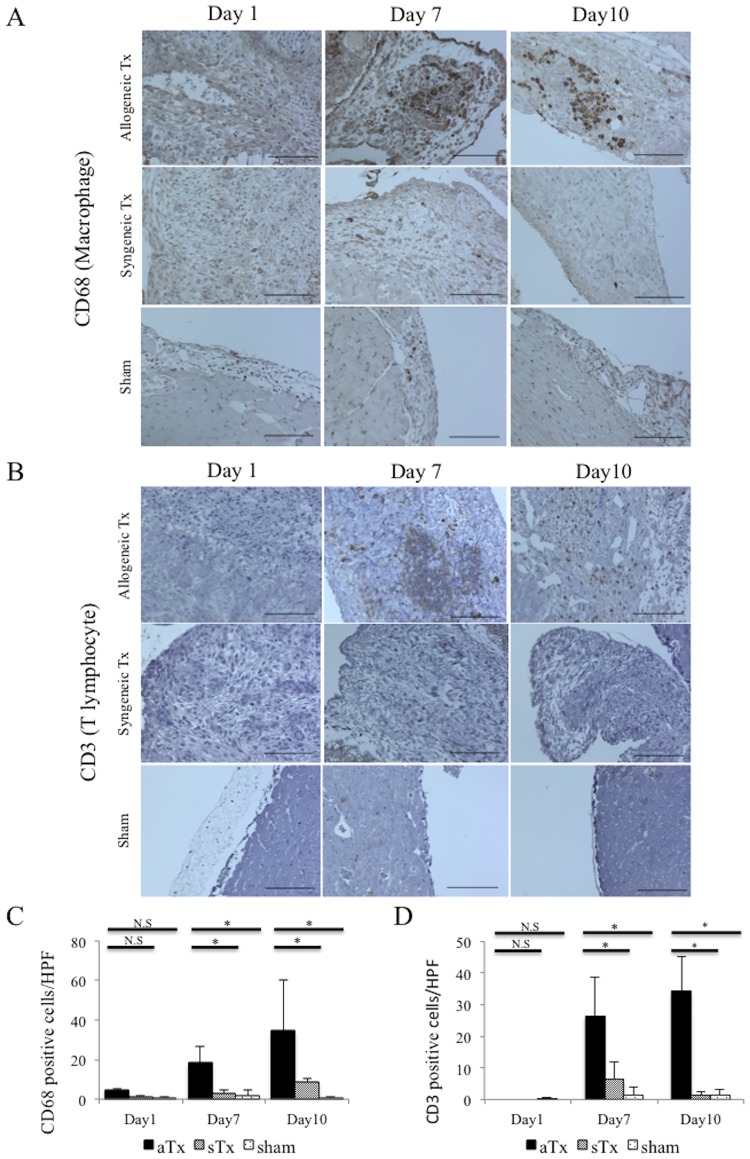
Infiltration of activated macrophages and T lymphocytes into the transplanted graft. A, infiltration of CD68 positive activated macrophages into the transplanted grafts in the allogeneic Tx group, the syngeneic Tx group, and sham group at days 1, 7, and 10. bar = 100μm; B, infiltration of CD3 positive T lymphocytes into the transplanted grafts in the allogeneic Tx group, the syngeneic Tx group, and sham group at days 1, 7, and 10. bar = 100μm; C, comparison of the infiltrating CD68 positive cell counts per HPF among the allogeneic Tx group, the syngeneic Tx group, and sham group at days 1, 7, and 10; D, comparison of the infiltrating CD3 positive cell counts per HPF among the allogeneic Tx group, the syngeneic Tx group, and sham group at days 1, 7, and 10 * indicates *P* <0.05; N.S, not significant.

Whole hearts hearts from all the groups were sampled for immunohistological examination following the PET-CT imaging studies at days 1, 7, and 10. Transverse sections at the mid-ventricular level that included the transplanted graft were immunohistolochemically labeled for CD68 and CD3. CD68-positive cells or CD3-positive cells were rarely detected in any of the groups at day 1. In contrast, at days 7 and 10, both CD68 and CD3-positive cells were densely present in the transplanted graft of the allogeneic Tx group, but not in the syngeneic or the sham groups (Fig 4A and 4B).

The percentages of CD68-positive macrophages and CD3-positive T-lymphocytes in the graft were low at day 1 and were not different among the 3 groups. In contrast, at days 7 and 10, the allogeneic Tx group, but not the syngeneic Tx or the sham groups, showed a significantly and markedly higher percentage of the CD68 positive macrophage (at day 7: allogeneic Tx group; 18.3±8.4 cells/HPF; syngeneic Tx group; 2.7±2.2 cells/HPF, P = 0.02; sham group; 1.9±2.7 cells/HPF, P = 0.02, and at day 10: allogeneic Tx group; 34.7±25.3 cells/HPF; syngeneic Tx group; 8.7±1.9 cells/HPF, P = 0.0495; sham group; 0.9±0.6 cells/HPF, P = 0.046); CD3-positive T-lymphocytes (at day 7: allogeneic Tx group;26.4±12.3 cells/HPF; syngeneic Tx group; 6.3±5.4 cells/HPF, P = 0.037; sham group; 1.3±2.5 cells/HPF, P = 0.01; at day 10: allogeneic Tx group; 34.3±10.8 cells/HPF; syngeneic Tx group; 1.3±0.9 cells/HPF, P = 0.495; sham group; 1.2±2.1 cells/HPF, P = 0.046) (Fig 4C and 4D).

### Predominant expression of TSPO and associated proteins subsequent to allogeneic cell transplantation

**Fig 5:**
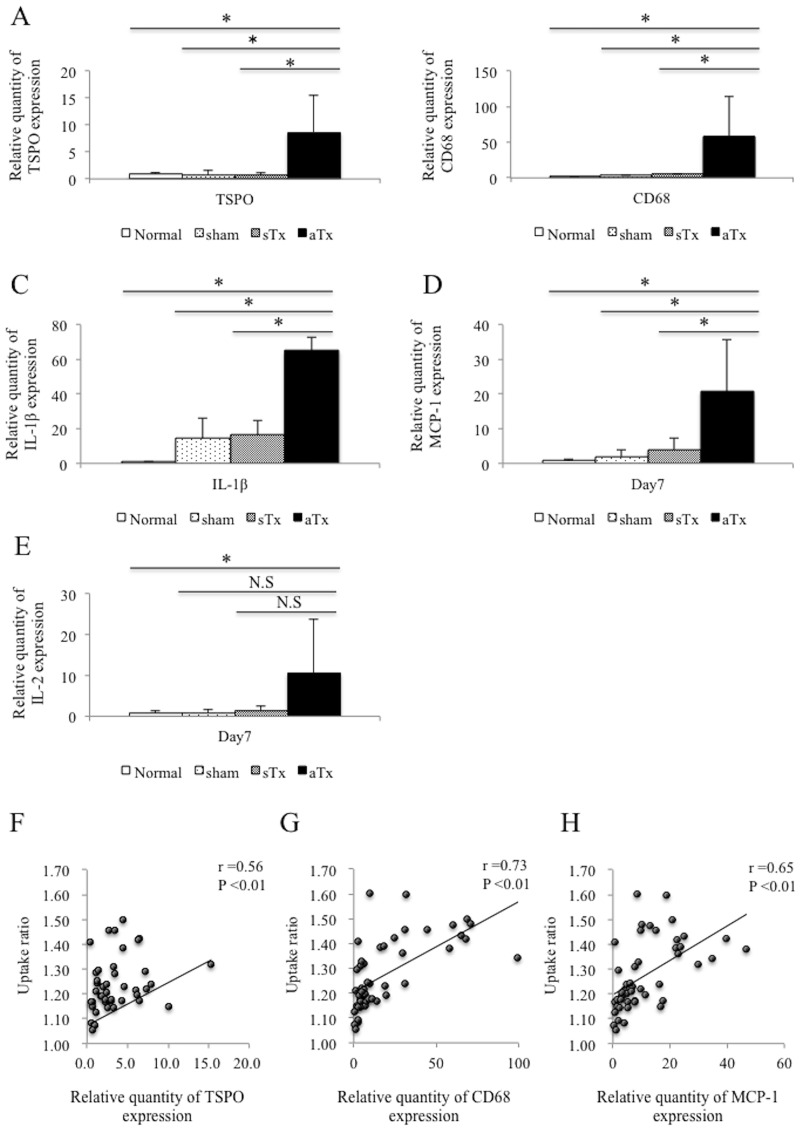
Expression of TSPO and associated proteins after iPSC-cardiac sheet transplantation. A-E, serial changes in the expression of *TSPO*, *CD68*, *IL-1beta*, *MCP-1* and *IL-2* in the allogeneic Tx group, the syngeneic Tx group, and sham group at day 7 after transplantation; F-H, correlation between the uptake ratio and expression of *TSPO*, *CD68* and *MCP-1; TSPO* indicates translocator protein; *IL-1*, interleukin-1; iPSC, induced pluripotent stem cells; Tx, transplantation; LV, left ventricle; * indicates *P* <0.05; N.S, not significant.

Expression of *TSPO*, which is the target of PET tracer to detect inflammation, and that of associated inflammation-related genes was assessed by real-time PCR. Expression of these genes was not significantly different among the 3 groups at day 1. The allogeneic Tx group displayed a significantly higher *TSPO* expression than the syngeneic Tx and the sham groups at day 7 (Fig 5A). Consistent with this finding, expression of *CD68*, *IL-1*beta, and *MCP-1* was significantly higher in the allogeneic Tx group than in the syngeneic Tx and the sham groups at day 7 (Fig 5B–5D). In contrast, expression of the proteins was not significantly different over the study period in the syngeneic Tx and the sham groups. Furthermore, the uptake ratio, assessed by the PET imaging study, was significantly correlated with the expression of *TSPO*, *CD68* and *MCP-1* (r = 0.56, *P* <0.01, r = 0.73, *P*<0.01 and r = 0.65, *P*<0.01, respectively, Fig 5F–5H). CD68 positive cells in the transplanted graft also expressed TSPO in the allogeneic Tx group at day 7 (S2 Fig).

### Bioluminescence imaging-based analysis of graft survival

**Fig 6:**
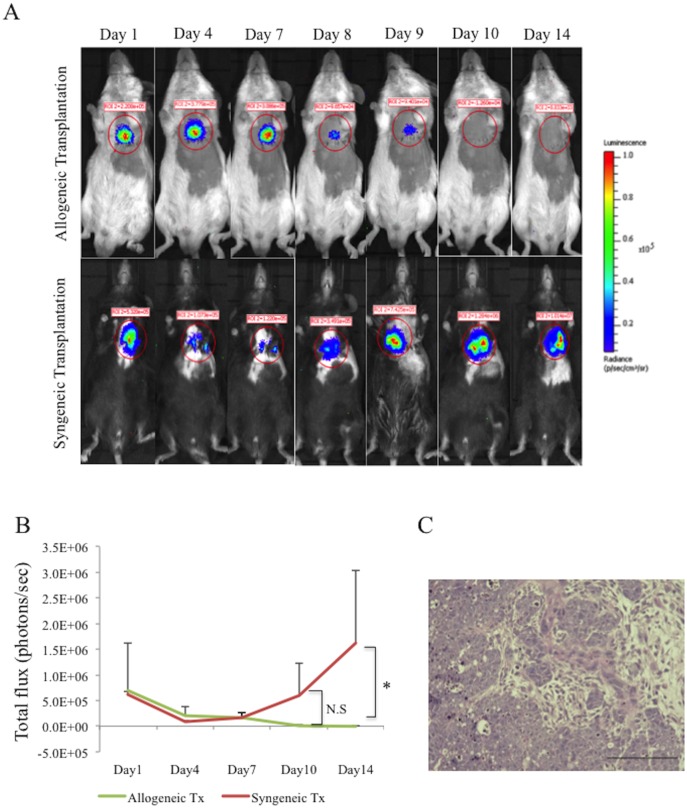
Bioluminescence imaging. A, serial evaluation of transplanted graft survival by bioluminescence imaging in the allogeneic and syngeneic Tx groups; B, quantitative comparison of bioluminescence between the allogeneic and syngeneic Tx groups; C, teratoma formation in the syngeneic Tx group at day 7, bar = 100μm; Tx indicates transplantation; iPSC, induced pluripotent stem cells; LV, left ventricle; * indicates P <0.01; N.S, not significant.

Survival of the grafted cells that expressed luciferase was serially assessed in the allogeneic Tx and the syngeneic Tx groups by using BLI. Strength of the bioluminescence declined over 9 days in the allogeneic Tx group, and was not detectable at day 10. In contrast, bioluminescence in the syngeneic Tx group persisted, showing an increase over time, consistent with teratoma formation. (Fig 6A–6C)

### Phenotype change of the macrophages accumulated in the rejected allograft

**Fig 7:**
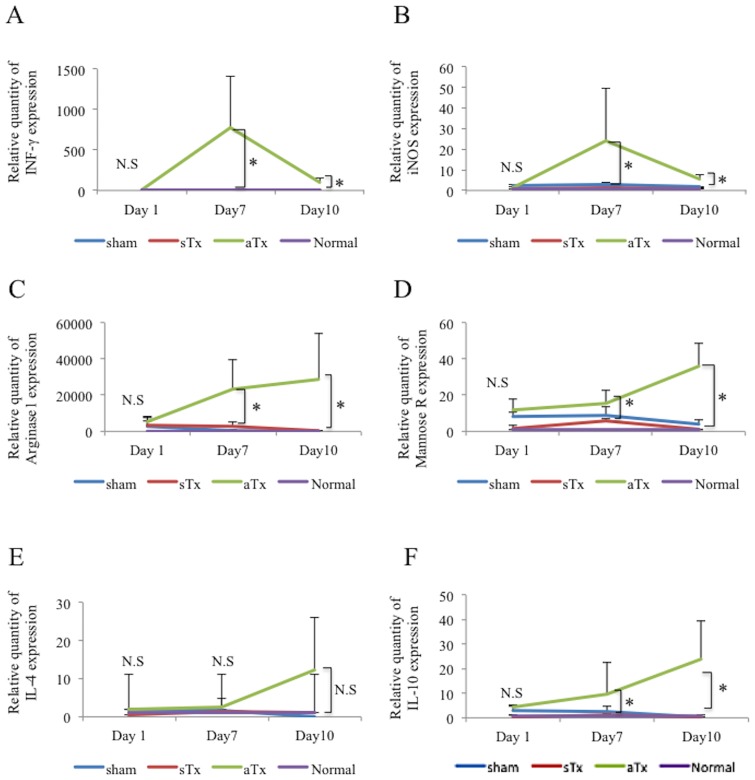
Phenotype change of the macrophages accumulated in the rejected allograft. A-F, serial changes in the expression of *IFN-gamma*, *iNOS (NOS2)*, *Arginase-1*, *Mannose receptor*, *IL-4* and *IL-10* in the allogeneic Tx group, the syngeneic Tx group, sham group and non-surgical control group at days 1, 7 and 10 after transplantation; *INF-gamma* indicates interferon gamma; *iNOS*, inducible nitric oxide synthase; Tx, transplantation; iPSC, induced pluripotent stem cells; LV, left ventricle; * indicates *P* <0.05; N.S, not significant.

Expression of genes associated with the phenotype of the macrophage, such as *iNOS*, *IFN-gamma*, *Arg1*, *Marc1*, *IL-4*, and *IL-10*, in the heart was assessed by real-time PCR. Expression of these genes was not significantly different among the 3 groups at day 1. While the syngeneic Tx and the sham groups displayed minimum expression of thesegenes throughout the study period, the allogeneic group displayed an interesting expression pattern (Fig 7A–7F). Expression of *iNOS* and *INF-gamma*, which are the reported markesr of M1 type macrophage and its stimulating factor, respectively, was modestly higher at day 7 than at days 1 and 10. In contrast, expression of *Arg1*, *Marc1*, *IL-4* and *IL-10*, which are the reported markers of M2 type macrophage and its stimulation factors, were significantly or modestly higher at day 10 than that at days 1 and 7.

## Discussion

This study explored the utility of ^18^F-DPA-714 PET imaging for the detection of immunological rejection of the allogeneic iPSC-cardiac cell transplants in a murine model. Luciferase-expressing cardiac graft obtained by inducing cardiomyogenic differentiation of murine iPSCs, containing 90% cardiac troponin T positive cells, was transplanted over the LV surface of the syngeneic or the allogeneic murine heart. Tail vein injection of ^18^F-DPA-714 resulted in physiological distribution of the radioisotope, which was visualized and quantified by the PET-CT scanning, the results of which were consistent with the autoradiography counts of the radioisotope that was accumulated in the graft. Allogeneic transplantation of the cardiac graft over the LV surface, but not syngeneic one, induced accumulation of the radioisotope signals at days 7 and 10, when CD68 positve macrophages and CD3 positive T-lymphocytes were densely accumulated in the allogeneic graft, but not in the syngeneic graft, as assessed by immunohistochemical study. In addition, bioluminescence study to quantitate the survival of luciferase-expressing graft demonstrated disappearance of the grafted cells by day 10 after the allogeneic transplantation. Interestingly, factors associated with M1 macrophages were upregulated at day 7 and those with M2 macrophages were upregulated at day 10 after the allogeneic transplantation.

This study was conducted to develop a clinically applicable imaging technology to assess immune rejection of allogeneic cell transplants, using the murine model. T-lymphocytes are assumed to play a critical role in the immune response against the allogeneic cell transplants. This study examined the TSPO-positive activated macrophages in the graft. It is known that activated macrophages are involved in innate and acquired immune response as a scavengers and antigen-presenting cells to stimulate the T-lymphocytes to initiate acquired immune response [18, 19]. This study showed that both CD68-positive macrophages and CD3-positive T-lymphocytes were accumulated in the same regions of the grafted tissues, indicating the positive crosstalk between them to accelerate the immune response mediated inflammation in the tissue. Macrophage targeting magnetic resonance imaging or nanoparticle PET techniques have been developed as experimental tools to detect immune response, though they were not established for the clinical use [20, 21]. In contrast, ^18^F-DPA-714 is in clinical use to detect inflammation accompanying cerebral infarction [22, 23], suggesting the potential application of this imaging tool in the cell transplantation therapy area.

Since TSPO is present in the outer membrane of the mitochondria, TSPO-targeted imaging may theoretically not be sensitive in the tissue in which mitochondria is inherently abundant, such as cardiac tissue. However, there are actually multiple reports in which single-photon emission computed tomography (SPECT) imaging targeting TSPO was feasible and sensitive in the myocardial inflammation [24]. In addition, in this study, allogeneic iPSC-derivatives were transplanted into the dorsal side of subcutaneous tissue, which is not rich in mitochondria, as a positive control to explore the feasibility and sensitivity of TSPO-targeting PET imaging for cardiac tissue. There was a significant correlation between SUVmax by the PET imaging and the radioactivity counts by autoradiography in the excised cardiac tissue, similar to the subcutaneous tissue, indicating the feasibility and sensitivity of TSPO-targeted PET imaging in the murine cardiac tissue. Furthermore, experiments comparing the allogeneic and the syngeneic transplantations clearly delineated the difference in TSPO signals by PET imaging, indicating that the TSPO-targeted PET imaging was feasible and sensitive in detecting macrophage mediated inflammation in response to allogeneic transplantation in the cardiac tissue.

It is possible that the accumulation of TSPO-positive macrophages was non-specific, resulting from the cell/tissue transplantation-related inflammation or innate immune response. However, at day 1 when non-specific inflammation or innate immune response would be at its peak [25], CD68 macrophages were rarely present in the graft and expression of inflammatory cytokines, such as IL-1beta, was not upregulated in any of the 3 groups, indicating that the transplantation method in this study of simply placing the scaffold-free cell clusters over the heart surface, evokes minimum non-specific, cell/tissue transplantation-related inflammation or innate immune response.

The initial drop in luciferase activity observed in this study in both the syngeneic and allogeneic models was considered to have resulted from the decrease in the survival of luciferase-carrying cells due to initial ischemia or malnutrition immediately after transplantation on to the cardiac surface. It is possible that non-specific inflammation affected the intensity of bioluminescence. However, strong BLI signal was observed in the allografted animal at day 8, but the BLI signal at day 10 was lower despite persisting inflammation, suggesting that the effect of non-specific inflammation on BLI signal would be possible but minimum.

Dynamic change in the expression of the macrophage phenotype-related factors was detected in the heart that was subjected to the allogeneic cell transplantation between days 7 and 10, indicating macrophage polarization during this time-period. M1 macrophage related factors were prominent at day 7, while M2 macrophage related ones were prominent at day 10. Classically activated macrophages (M1 type macrophage) are activated by IFN-gamma and via toll-like receptor to produce a large amount of iNOS, resulting in cytotoxicity, while alternatively activated macrophages (M2 type macrophage) are activated by IL-4 or IL-13 to produce IL-10, repairing tissues and functioning as an immunosuppressive immune cells [26]. This phenomenon was consistent with the survival pattern of the graft in our study, as assessed by BLI, in which the luciferase-positive cells were present at day 7, and were absent at day 10. It was thus considered that immune response against the transplanted allogeneic cells was converged by day 10 when the antigenic cells disappeared. This finding has important implications for clinical use of this imaging tool. The monitoring of the survival of the transplanted cells, which is known to be closely correlated with the therapeutic effects or complications such as teratoma formation, has been poorly established. ^18^F-DPA-714 PET imaging might predict the persistence of the cells that are involved in the macrophage mediated host immune response.

This study is limited by the use of murine experimental model. It might be argued that a single cell-line of the iPSCs is insufficient to draw valid conclusions. However, the 959A2-1 cell-line used in this study has been well investigated after cardiomyogenic differentiation in a number of studies and yielded consistent results. Nonetheless, further study using human iPSCs transplanted into large animal models may be needed for preclinical proof-of-concept. Future studies using human iPSCs for large animal models would address another potential limitation of this study, which is the difference in immune response to allogeneic antigens between human and murine. Lungs intrinsically display a high uptake of DPA-714 because of the alveolar macrophages activated in response to the exposure to external micro-organisms. However, we considered that the high intensity area in the lungs in this work represented alveolar macrophages activated by thoracotomy-related atelectasis and hence those areas should be excluded from radioactivity measurement. Using healthy mice is also one of the limitations of this work. Further studies using a murine model of infarcted heart or clinical heart failure controls would be needed to optimize the TSPO-targeting PET imaging for allogeneic iPS cell therapy for the heart. A number of studies using murine allogeneic organ transplantation model, investigating the immune response to allogeneic grafts have been reported. The results described in these reports, suggest that the findings of the current study directly reflect the clinical situation to some degree.

## Conclusions

In conclusion, the ^18^F-DPA-714-PET imaging study enabled the quantitative visualization of activated macrophages-mediated immune rejection of the allogeneic transplant of iPSC-cardiac sheet. This imaging tool may contribute to the understanding and monitoring of host-immune response in the allogeneic cell transplantation therapy.

## Supporting Information

S1 Fig Radio synthesis of ^18^F-DPA-714. (TIFF)

S2 Fig Immunohistochemical staining of the allogeneic iPSC-CM sheet transplant at day 7. Transplanted iPSC-CMs sheets stained with anti-CD68 antibody (Alexa Fluor 488), anti-TSPO antibody (Alexa Fluor 647) and DAPI, were analyzed by confocal laser scanning microscopy; bar = 20μm.
(TIFF)
