# Supplementary figures and images for: Development of PET Imaging to Visualize Activated Macrophages Accumulated in the Transplanted iPSc-Derived Cardiac Myocytes of Allogeneic Origin for Detecting the Immune Rejection of Allogeneic Cell Transplants in Mice
Source: PLoS One. 2016 Dec 8;11(12):e0165748. doi: 10.1371/journal.pone.0165748 (PMC5145152; doi:10.1371/journal.pone.0165748)

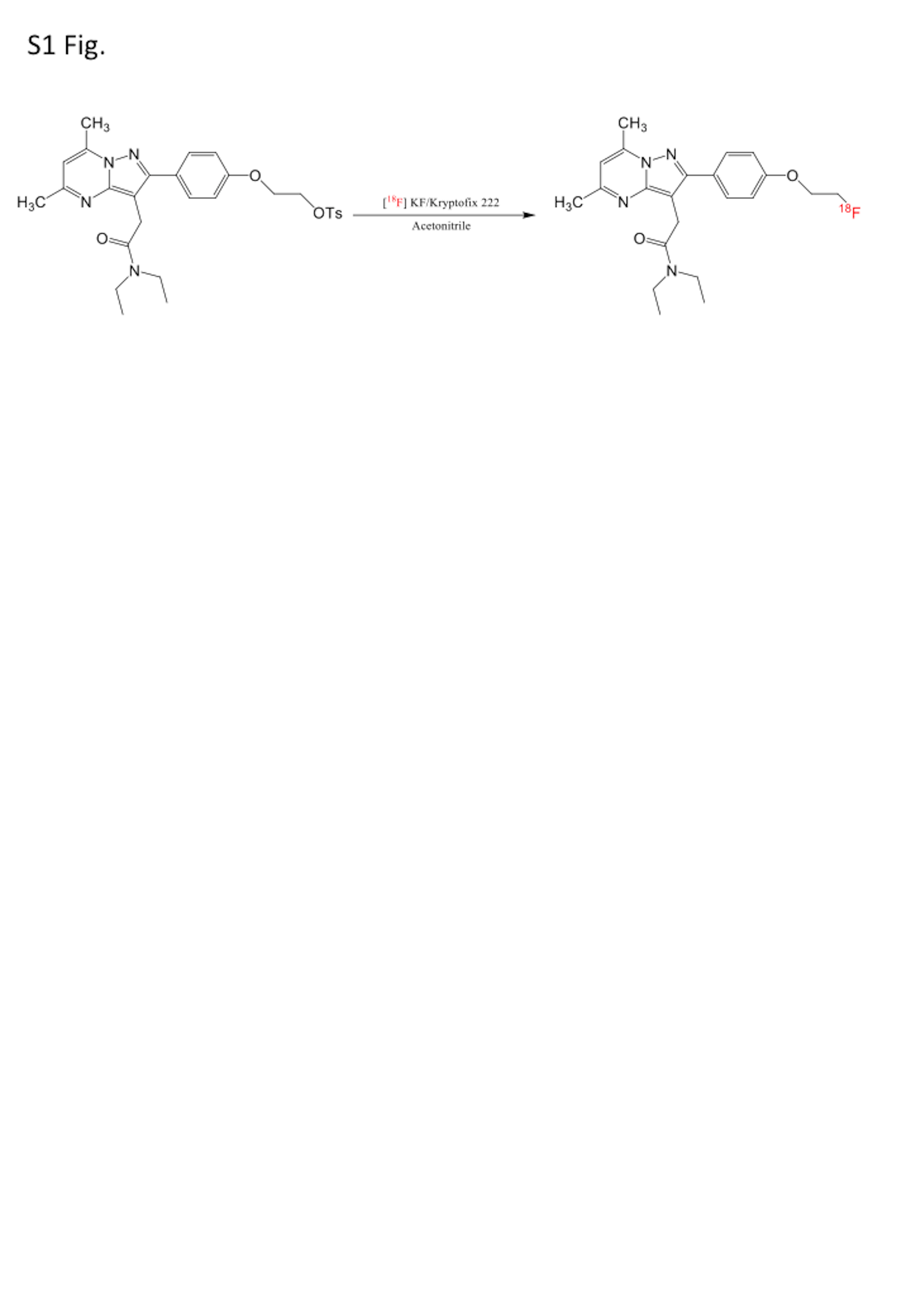

Supplement: S1 Fig — (TIFF) [file pone.0165748.s001.tiff]

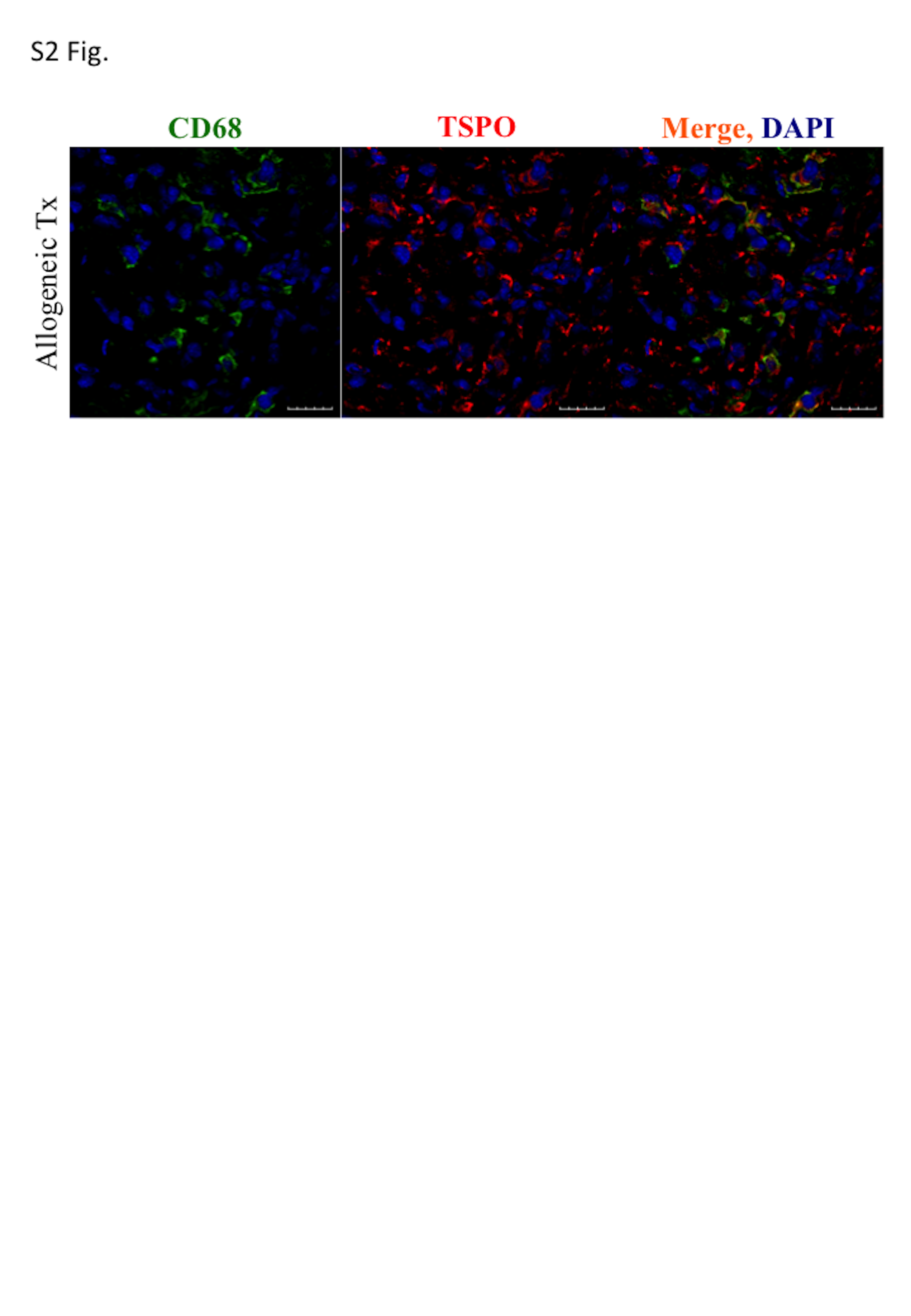

Supplement: S2 Fig — Transplanted iPSC-CMs sheets stained with anti-CD68 antibody (Alexa Fluor 488), anti-TSPO antibody (Alexa Fluor 647) and DAPI, were analyzed by confocal laser scanning microscopy; bar = 20μm. (TIFF) [file pone.0165748.s002.tiff]
